# Short-term hearing aid use reduces auditory cortical responses to speech-in-noise listening among older adults with age-related hearing loss

**DOI:** 10.3389/fnagi.2026.1690956

**Published:** 2026-04-16

**Authors:** Katherine M. Becker, Patrice Voss, Zaida Escila Martinez-Moreno, Francois Prévost, Anthony Zeitouni, Alejandro Lopez Valdes, Etienne de Villers-Sidani

**Affiliations:** 1 Department of Neurology and Neurosurgery, Montreal Neurological Institute, McGill University, Montreal, QC, Canada; 2 Department of Audiology and Speech-Language Pathology, McGill University Health Center, Montreal, QC, Canada; 3 Department of Otolaryngology-Head and Neck Surgery, McGill University, Montreal, QC, Canada; 4 Trinity College Institute of Neuroscience, Trinity College Dublin, Dublin, Ireland; 5 Global Brain Health Institute, Trinity College Dublin, Dublin, Ireland; 6 Department of Electronic and Electrical Engineering, School of Engineering, Trinity College Dublin, Dublin, Ireland; 7 Trinity Center for Biomedical Engineering, Trinity College Dublin, Dublin, Ireland

**Keywords:** age-related hearing loss (ARHL), hearing aids (HA), hearing loss, magnetoencephalography (MEG), speech in noise (SIN)

## Abstract

Age-related hearing loss (ARHL) is an increasingly common feature of aging and has been linked to poorer psychosocial wellbeing and increased dementia risk. Individuals with ARHL experience speech perception difficulties in noisy environments, wherein the brain must “turn up the volume” or upregulate neural activity to accurately parse speech from background noise. As the aging population steadily increases worldwide, it is essential to develop effective, non-invasive, and easily attainable interventions to reduce the societal impact of ARHL. Although hearing aids (HAs) have been proposed as an effective means to remediate or mitigate ARHL-related speech-in-noise perception, relatively few studies have investigated the short-term impact of HAs on brain function. In this preliminary study, we used magnetoencephalography to investigate the neurophysiological effects of short-term HA use in individuals with ARHL during a speech-in-noise (SIN) task. Results revealed significant reductions in activity in right-hemisphere regions after only 3 months of regular daily HA use in first-time users. In contrast, we found that individual differences in SIN performance change were best explained by the magnitude of reductions in left-hemisphere activity following short-term hearing aid use. Taken together, these findings suggest that auditory cortical responses during speech-in-noise listening can be rapidly modulated by short-term hearing aid use, providing evidence for experience-dependent plasticity in older adults with age-related hearing loss.

## Introduction

Presbycusis, or age-related hearing loss (ARHL), is ubiquitous in human aging, affecting more than 65% of adults by the age of 70 years (Goman and Lin, 2016; Lin et al., 2011) and steadily increasing to more than 80% of individuals aged 85 and above (Lin et al., 2011; Wattamwar et al., 2017). Hearing loss has been cited as the third most common cause of years-lived-with-disability (YLD) worldwide and is the leading cause of YLD in individuals aged 70 years and above (Haile et al., 2021). Difficulties in parsing speech from background noise are frequently reported by individuals with ARHL (Peters et al., 1998; Pichora-Fuller and Souza, 2003; Sheldon et al., 2008; Humes et al., 2012; Slade et al., 2020) and are cited as the greatest inconvenience to living with a hearing impairment (Jorgensen and Novak, 2020). Chronic undersampling of the speech waveform may create a noisy neural representation with low fidelity to the original signal, making it substantially more difficult to suppress background noise in challenging listening environments (Lopez-Poveda and Barrios, 2013; Lopez-Poveda, 2014). A decrease in signal-to-noise ratio during speech recognition tasks may thus cause older adults to “turn up the volume” or upregulate brain activity to inhibit irrelevant information, as evidenced by increased neural activity (Grossman et al., 2002; Wong et al., 2009; Du et al., 2016), neural synchrony (Ross et al., 2020), speech-brain entrainment (Decruy et al., 2019, 2020; Fuglsang et al., 2020; Schmitt et al., 2022), and inter-regional connectivity (Bidelman et al., 2019; Price et al., 2019; Vander Ghinst et al., 2021) during speech-in-noise (SIN) tasks.

However, the functional interpretation of these neural increases remains an area of active debate. Although such findings are often discussed within the framework of the neural compensation hypothesis (Reuter-Lorenz and Cappell, 2008), similar cortical hyper-responsivity has also been observed in older adults in the absence of background noise or linguistic content (Herrmann et al., 2016; Presacco et al., 2016), suggesting that these effects may reflect early-stage sensory changes related to aging or hearing loss rather than compensatory cognitive mechanisms per se. Importantly, age-related auditory changes appear to differ across levels of the auditory pathway: while cortical responses to auditory stimuli are frequently reported to be enhanced in older adults, subcortical phase-locked responses are often reduced, highlighting the complexity of neural reorganization associated with aging and hearing loss. Since aging and hearing loss are highly correlated in older populations, disentangling their respective contributions to these neural effects remains challenging and is an ongoing topic of investigation.

Several studies examining auditory processing in older adults have reported increased neural responses during speech perception (Wong et al., 2009; Manan et al., 2013; Vaden et al., 2015; Presacco et al., 2016; Presacco et al., 2019; Decruy et al., 2019; Kuruvilla-Mathew et al., 2022), with increased neural activity being associated with poorer performance in SIN tasks by older adults with ARHL (Glick and Sharma, 2020). In contrast, a handful of neuroimaging studies have reported decreased neural activity after HA treatment (Hwang et al., 2006; Alain et al., 2010; Glick and Sharma, 2020), potentially reflecting reduced processing demands following auditory amplification. In these studies, reductions in neural activity have primarily been observed in auditory and frontal cortical regions during speech perception tasks and have been interpreted as reflecting reduced processing demands following auditory amplification. However, several other studies have also reported increased neural activity after HA use (Hwang et al., 2006; Vogelzang et al., 2021; Karawani et al., 2022). These increases have been reported across different cortical regions and task contexts, such as sentence processing and speech perception in quiet or noise, underscoring the heterogeneity of observed neural effects following hearing aid use. This directional discrepancy may stem from differences in selected regions of interest (frontal versus temporal areas), experimental design (complex sentences presented in noise or silence), or duration of HA treatment. Additional evidence for attenuation of neural activity after hearing augmentation comes from recent pediatric studies using magnetoencephalography (MEG), which showed that the duration of HA use was negatively correlated with neural synchrony, defined as task-related oscillatory synchronization reflected by phase-locked or power-based activity within specific frequency bands, during a working memory task (Heinrichs-Graham et al., 2022a, 2022b). Together, these mixed findings underscore the need for longitudinal studies that carefully characterize how hearing aid use modulates neural responses across tasks, brain regions, and developmental stages, particularly in older adults with ARHL. Although numerous studies have examined neural responses to speech perception and the effects of hearing aid use, it remains unclear how initial hearing aid use influences longitudinal changes in cortical response magnitude during speech-in-noise listening in older adults. In this context, hearing aid use can be conceptualized as a sustained alteration of auditory input over daily listening, providing a basis for examining such changes over time.

The goal of this study was to characterize within-subject, longitudinal changes in cortical responses during speech-in-noise listening following short-term hearing aid use in first-time users under conditions in which task performance was explicitly matched across sessions. Magnetoencephalography (MEG) was selected for this study because its millisecond temporal resolution is well-suited for capturing rapid cortical dynamics during speech-in-noise listening and because it allows source-level characterization of auditory cortical responses without reliance on hemodynamic measures. We hypothesized that older adults with ARHL would exhibit reduced normalized MEG magnitude after 3 months of HA use in auditory and perisylvian cortical regions known to support speech perception. Given the behavior-matched design, which aimed to equate task performance across sessions, these neural changes were expected to reflect differences in neural responses rather than behavioral success or task performance. Moreover, we anticipated that the magnitude of these neural changes would vary as a function of acoustic context (cocktail noise versus connected speech) and trial outcome (correct versus incorrect), which were examined descriptively to characterize context-dependent patterns rather than through direct statistical contrasts. Finally, we anticipated that these effects would be primarily lateralized to the right hemisphere, as several studies have indicated that degraded auditory input may shift activity to the right hemisphere (Shtyrov et al., 1998; Shtyrov et al., 2000; Kujala et al., 2002; Sequeira et al., 2008).

## Methods

### Participants

**Figure 1:**
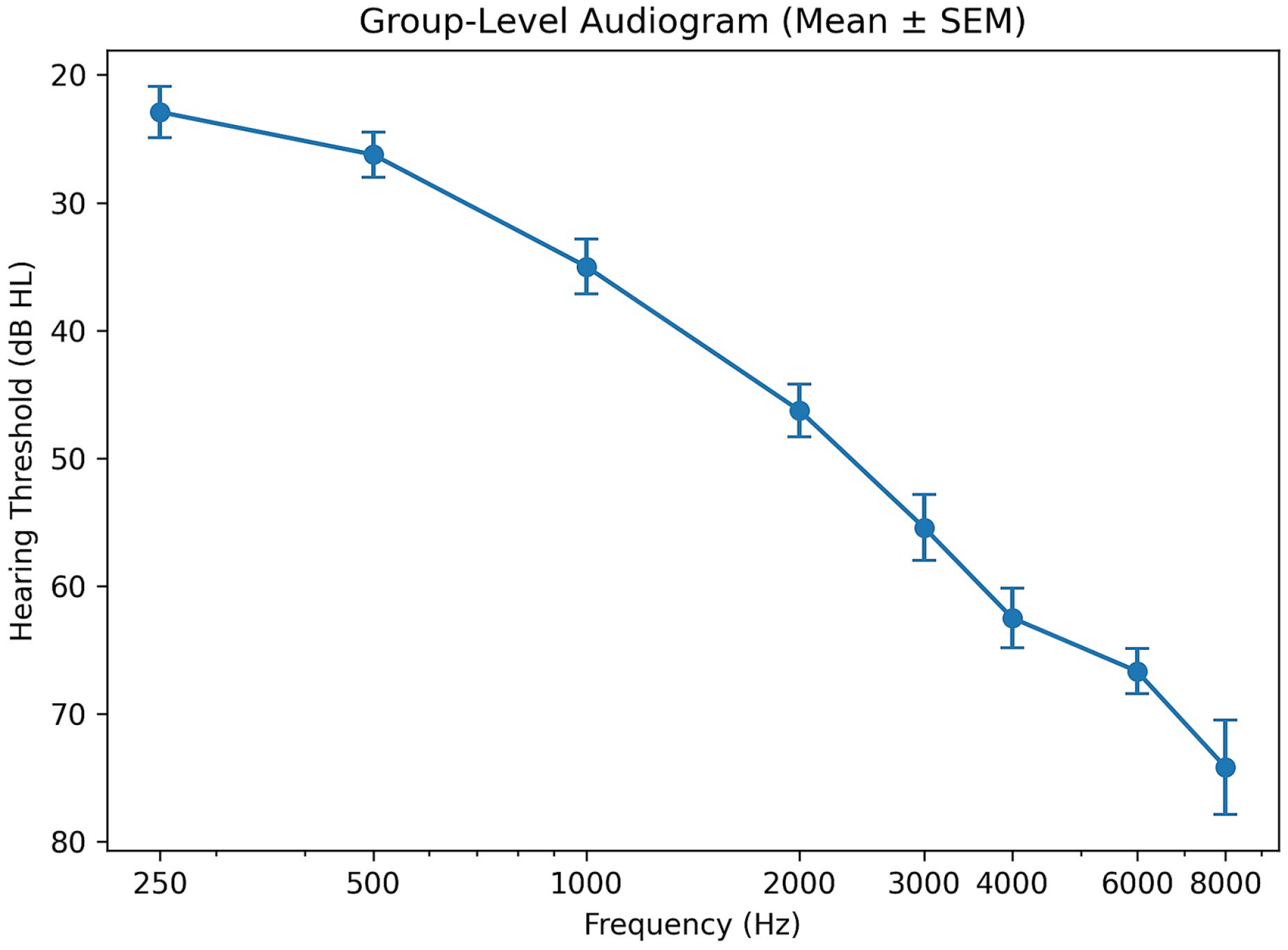
Group-level audiogram showing mean pure-tone hearing thresholds (dB HL) across standard audiometric frequencies prior to hearing aid fitting. Thresholds are pooled across ears and averaged across participants; error bars denote the standard error of the mean (SEM). Lower positions on the *y*-axis indicate poorer hearing sensitivity.

Twelve older adults (6 men; mean age = 75.8 years, SD = 6.7) with ARHL participated in the current study. A thirteenth recruited participant was excluded due to abnormally high normalized MEG magnitude (ROI z-scores greater than 2.5 standard deviations above the mean), suggestive of a non-representative signal (e.g., residual artifact or atypical normalization) that could disproportionately influence group-level analyses. Audiological measures were obtained by a licensed audiologist. All participants had an average pure-tone threshold exceeding 35 dB of normal hearing for frequencies between 0.25 and 8 kHz, with a maximum slope of 20 dB/octave between 1 and 4 kHz. This audiometric profile is characteristic of age-related hearing loss and is summarized at the group level in a pre–hearing-aid audiogram (Figure 1). Additionally, subjects exhibited no reverse slope (maximum −5 dB/octave) between 0.25 and 1 kHz and symmetrical hearing loss (maximum 10 dB average difference between ears). Potential participants whose impairment was related to noise-induced occupational hearing loss or tinnitus were excluded from the study. All participants were not cognitively impaired [screened with the Montreal Cognitive Assessment (MoCA)] and had no history of neurological or psychiatric conditions. All participants were fitted with bilateral Oticon hearing aids (Oticon Inc., Somerset, New Jersey, USA) by a licensed audiologist and were instructed to wear them at least 8 h/day for 3 months. Hearing aids were programmed by a licensed audiologist using standard clinical fitting procedures. Individualized amplification was determined using prescriptive targets based on each participant’s audiogram, with dynamic compression and manufacturer-recommended noise-reduction functions configured according to clinical defaults. All procedures were approved by the institutional review board of the Montreal Neurological Institute, and all participants provided written informed consent.

### Experimental setup

Magnetoencephalogram data were acquired on a CTF MEG 275 gradiometer system (CTF MEG Neuro Innovations, Inc., Coquitlam, BC, Canada) as participants completed four HINT runs (hearing-in-noise tasks; Nilsson et al., 1994) while seated in the MEG. During these runs, participants were asked to repeat 20 short sentences embedded in multispeaker babble noise created from four speakers (two female) in either French (Vaillancourt et al., 2005) or English (Nilsson et al., 1994), depending on the subject’s native language. The HINT sentences consisted of naturally spoken speech produced by a single talker per language, and the babble noise reflected naturalistic multi-talker speech rather than acoustically parameterized stimuli. Fundamental frequency characteristics were not explicitly manipulated and were kept constant across pre- and post–hearing-aid sessions.

Since hearing aids can interfere with the detection of magnetic fields, participants did not wear hearing aids during MEG acquisition. Instead, auditory stimuli were delivered via a custom setup developed by the Eriksholm Research Centre, which is part of Oticon, the developer of the hearing aids. Before being delivered to the participant through Etymotic ER-3A insert earphones with foam tips (Etymotic Research) at approximately 73 dB, stimuli were filtered through a single hearing aid positioned within a portable anechoic box. This hearing aid was programmed using the same individualized amplification settings matched to each participant’s clinically fitted devices, based on their audiogram and standard fitting procedures.

Two initial adaptive staircase runs were used to establish two individual signal-to-noise (SNR) hearing thresholds corresponding to 50 and 80% success rates for correctly repeated words over the entire 20-sentence runs. Participants’ performance was scored on a word-by-word basis. The subsequent two runs were presented with fixed SNRs, with values that were specific to each individual, as determined during the 50 and 80% staircase runs. The adaptive staircase runs served only to determine individualized SNRs for the subsequent fixed SNR conditions and were not designed to assess longitudinal changes in speech perception performance.

During these two fixed SNR runs (henceforth referred to as the 50% SNR and 80% SNR conditions), in addition to the stimuli being sent through the physical hearing aid described above, they were first digitally altered by a proprietary novel noise-reduction algorithm (provided by Oticon) intended to mimic the HA’s built-in noise-reduction algorithms. Each sentence trial began with 3 s of initial multi-speaker babble noise, followed by a semantically meaningful sentence (ranging from 1.135 to 2.948 s in length) overlaid with the noise. An additional 1-s of noise was presented after each sentence, and participants were then asked to repeat the sentence as accurately as possible after the offset of the second noise period.

During the 50% SNR and 80% fixed-SNR conditions, any incorrect or omitted words resulted in the trial being labeled as “incorrect” for subsequent analyses.

### MEG acquisition

Magnetoencephalographic data were acquired using a whole-head CTF MEG 275-channel axial gradiometer system (CTF MEG Neuro Innovations, Inc., Coquitlam, BC, Canada) housed in a magnetically shielded room. Data were sampled at 12 kHz, with online anti-aliasing filters applied according to system defaults. Head position was continuously monitored throughout acquisition to maintain data quality, ensure analysis accuracy, and minimize artifacts.

### Data analysis

Data were preprocessed and analyzed in Brainstorm (Tadel et al., 2011), which is documented and freely available for download online under the GNU General Public License.^1^ Preprocessing and analysis followed good practice guidelines (Gross et al., 2013; Tadel et al., 2019), wherein eye, cardiac, and muscle artifacts were identified and projected from the data using signal space projectors. Bad trials and channel identification were aided via the use of an automated artifact rejection toolbox (Weisman et al., in preparation). The data were then manually inspected for any remaining artifacts not subtracted by the projectors or artifact rejection toolbox and removed from the data before importing and epoching.

Individual trials were separated into two periods to elucidate the mechanisms underlying noise suppression during periods with and without a speech signal of interest. These periods were defined as a ‘Noise’ (0.0–1.125 s) and ‘Sentence’ (3.0–4.125 s) epoch. Channels were standardized across participants and averaged in the time domain by trial type (correct or incorrect) across the On50 and On80 conditions to increase the total number of trials used in the analysis. Both correct and incorrect trials were analyzed separately. Head models were calculated using individual subject structural MRIs. Noise covariance matrices were computed using an empty-room recording acquired before each participant’s MEG session.

Sensor-level trial averages were first computed separately for each condition and trial type. These sensor-level averages were then source localized using the linear dynamic Statistical Parametric Mapping (dSPM) inverse operator implemented in Brainstorm (Dale et al., 2000), spatially smoothed (3 mm), and baseline-normalized to the −0.25 to 0 s interval occurring immediately prior to the trial onset. Due to the small number of trials, subject averages were subsequently low-pass filtered at 20 Hz to minimize the contribution of high-frequency noise.

Neural responses were quantified at the source level by averaging normalized source amplitudes within the Noise and Sentence epochs prior to region-of-interest (ROI) extraction. Individual source-localized maps were then projected onto the default anatomy (Colin27_2016) for group comparisons. Subject-level scout values were extracted from each region of interest (ROI) for each epoch and trial type, and these values were then used in all subsequent statistical analyses. Group difference maps were created by averaging individual subject maps projected onto the default anatomy and then subtracting the post-HA group map from the pre-HA group map.

Eight bilateral brain areas located in the inferior frontal—pars opercularis, pars orbitalis, pars triangularis—and temporal regions—superior temporal gyrus, transverse temporal gyrus (henceforth referred to as Heschl’s gyrus), and supramarginal gyrus—regions were selected as ROIs for their known role in speech processing (*a priori*, defined according to the Desikan-Killiany atlas) and were included in subsequent analyses. Each subject served as their own control, and statistical analyses were performed in R (v4.1.1, R Core Team, 2021). Shapiro–Wilks tests using the Shapiro test function from the rstatix library (Kassambara, 2021) revealed that the activity in the majority of ROIs was non-normally distributed. Due to the non-normality of the dataset, we compared each ROI before and after HA treatment using a non-parametric equivalent to the traditional Student’s paired t-test and the one-tailed paired Wilcoxon signed-rank test, as implemented in the coin library (Hothorn et al., 2006) in R.

Tests were performed separately for each epoch (Noise or Sentence), trial type (correct or incorrect), and ROI. Matched-pairs rank biserial correlation coefficients (*rc*) were calculated to quantify the effect size of each comparison using the non-parametric Wilcoxon Paired RC function from the rcompanion library (Mangiafico, 2022), as recommended by King et al. (2018). Due to the preliminary nature of the present study and the small sample size, we opted not to use statistical corrections for multiple comparisons to avoid ruling out potential leads for future research. Accordingly, the analytical approach emphasizes within-subject comparisons and effect size estimation, and we focus our result interpretations on brain-wide trends rather than on specific single ROI test results, such as consistent findings across a given hemisphere or epoch type.

## Results

### Behavioral results

As expected, there were no significant differences in HINT performance in either the 50% SNR (*t*(10) = 0.218, *p* = 0.416) or 80% SNR (*t*(10) = 0.697, *p* = 0.251) conditions before and after HA use, as SNR thresholds were adapted to maintain constant task performance across recording sessions. Speech reception thresholds derived from the adaptive staircase procedures were used to calibrate task difficulty and were not considered longitudinal outcome measures.

### Neuroimaging results (MEG)

**Figure 2:**
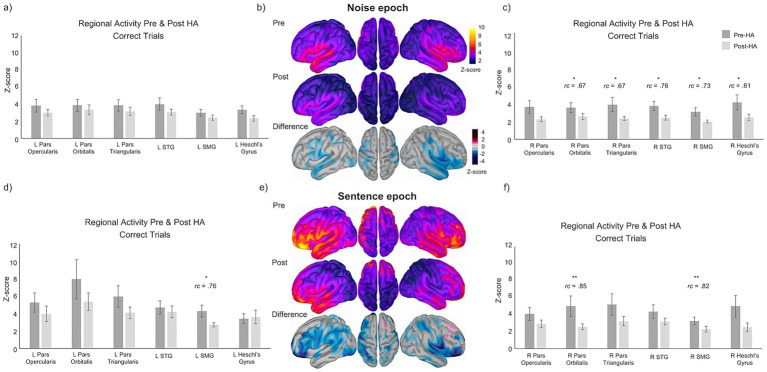
Source-localized images and bar graphs showing average group-level changes (*z*-scores) and standard error of the means for normalized MEG magnitude before and after 3 months of HA use by hemisphere for correct trials during the Noise and Sentence epochs (dark gray = pre-HA; light gray = post-HA). **(a)** Left hemisphere ROI group average z-scores for correct trials during the Noise epoch. **(b)** Unthresholded source localized group averages of activity before (top row) and after (second row) HA treatment. Z-score thresholded group average difference maps showing the change in neural activity before and after HA use (third row). **(c)** Right hemisphere ROI group average z-scores for correct trials during the Noise epoch. **(d)** Left hemisphere ROI group average z-scores for correct trials during the Sentence epoch. **(e)** Unthresholded source localized group averages of activity before (top row) and after (second row) HA treatment. Z-score thresholded group average difference maps showing the change in normalized MEG magnitude before and after HA use for the sentence epoch (third row). **(f)** Right hemisphere ROI group average z-scores for correct trials during the sentence epoch. Significance values are indicated by *p* < 0.05 = *, *p* < 0.01 = **.

Paired Wilcoxon signed-rank tests revealed that, overall, participants exhibited significantly decreased normalized MEG magnitude with moderate effect sizes (matched-pairs rank biserial correlation coefficients [*rc*]) after HA treatment for both correct and incorrect trials during the Noise and Sentence epochs. For all analyses, data from the 50%-SNR (On50) and 80%-SNR (On80) fixed-SNR conditions were averaged prior to statistical testing to increase the number of trials per condition. No direct statistical comparisons were conducted between the two SNR levels. Source-localized group average maps for correct trials are shown in the center column of Figure 2.

Correct and incorrect trials were analyzed separately to characterize patterns of normalized MEG magnitude changes across different conditions. No direct statistical comparisons were performed between correct and incorrect trials, and differences between trial types are therefore described descriptively rather than inferentially. Statistical significance was defined as a *p*-value of < 0.05. Where appropriate, results are additionally reported using conventional thresholds (e.g., *p* < 0.01, *p* < 0.001).

While there were no significant changes in activity for ROIs in the left hemisphere for the Noise epoch of correct trials (Figure 1a), several ROIs in the right hemisphere exhibited significantly reduced activity for correct trials with moderate effect sizes (Figure 1c). These areas included the right pars orbitalis (*Z* = −2.21, *p* < 0.05, rc = 0.67), pars triangularis (*Z* = −2.21, *p* < 0.05, rc = 0.67), superior temporal gyrus (STG; *Z* = −2.51, *p* < 0.05, rc = 0.76), supramarginal gyrus (SMG; *Z* = −2.41, *p* < 0.05, rc = 0.73), and Heschl’s gyrus (*Z* = −2.05, *p* < 0.05, rc = 0.61). Significant reductions in normalized MEG magnitude were also observed in several ROIs during the Sentence epoch of correct trials (Figures 1d,e) with large effect sizes in the left SMG (*Z* = −2.15, *p* < 0.05, rc = 0.76) and right SMG (*Z* = −2.70, *p* < 0.01, rc = 0.82), as well as in the right pars orbitalis (*Z* = −2.81, *p* < 0.01, rc = 0.85). For a complete list of Wilcoxon signed-rank results, see Supplementary Table 1.

**Figure 3:**
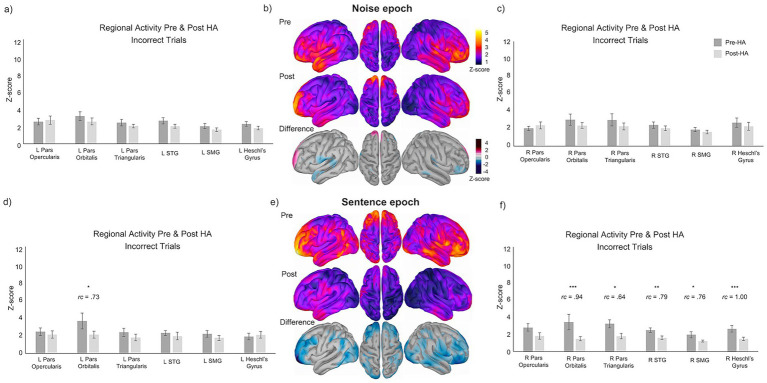
Source localized images and bar graphs showing average group-level changes (*z*-scores) and standard error of the means for normalized MEG magnitude before and after 3 months of HA use by hemisphere for incorrect trials during the Noise and Sentence epochs (dark gray = pre-HA; light gray = post-HA). **(a)** Left hemisphere ROI group average *z*-sco*res for incorrect trials during the Noise epoch.* **(b)** *Unthresholded source localiz*ed group averages of activity before (top row) and after (second row) HA treatment. *Z*-score thresholded group average difference maps showing the change in neural activity before and after HA use for incorrect trials (third row). **(c)** Right hemisphere ROI group average *z*-scores for incorrect trials during the Noise epoch. **(d)** Left hemisphere ROI group average *z*-scores for incorrect trials during the Sentence epoch. **(e)** Unthresholded source localized group averages of activity before (top row) and after (second row) HA treatment for incorrect trials. *Z*-score thresholded group average difference maps showing the change in normalized MEG magnitude before and after HA use for the Sentence epoch (third row). **(f)** Right hemisphere ROI group average *z*-scores for incorrect trials during the Sentence epoch. Significance values are indicated by *p* < 0.05 = *, *p* < 0.01 = **, *p* < 0.001 = ***.

There were no significant changes in ROI activity before and after HA use for the Noise epoch of incorrect trials. Conversely, six ROIs exhibited significantly reduced activity with moderate to large effect sizes during the Sentence epoch of incorrect trials: left pars orbitalis (*Z* = −2.41, *p* < 0.05, rc = 0.73), right pars orbitalis (*Z* = −3.18, *p* < 0.001, rc = 0.94), right pars triangularis (*Z* = −2.12, *p* < 0.05, rc = 0.64), STG (*Z* = −2.60, *p* < 0.01, rc = 0.79), right SMG (*Z* = −2.51, *p* < 0.05, rc = 0.76), and right Heschl’s gyrus (*Z* = −3.49, *p* < 0.001, rc = 1.00). For a complete list of Wilcoxon signed-rank test results for the paired incorrect comparisons, see Supplementary Table 2. Source localized group average maps for incorrect trials are displayed in the center column of Figure 3 for both the Noise (Figure 3b) and Sentence (Figure 3e) epochs, respectively.

## Discussion

Numerous studies have shown that peripheral hearing impairments have cascading effects on both the structure (Lin et al., 2011; Husain et al., 2011; Eckert et al., 2012; Boyen et al., 2013; Lin et al., 2014; Ren et al., 2018; Alfandari et al., 2018; Peelle et al., 2011; Eckert et al., 2019; Armstrong et al., 2020) and function (Peelle et al., 2011; Chen et al., 2016; Shilling-Scrivo et al., 2021; Fetoni et al., 2015; Fetoni et al., 2022; Wong et al., 2010) of the central auditory system. Despite this compelling body of evidence, the exact causal influence of age-related hearing loss (ARHL) on these neuropathophysiological changes has not been fully delineated (Slade et al., 2020; Jayakody et al., 2018; Mudar and Husain, 2016; Jafari et al., 2021; Wayne and Johnsrude, 2015; Uchida et al., 2019).

In the present exploratory study, we observed longitudinal reductions in normalized MEG magnitude following 3 months of hearing aid use in older adults with ARHL during a speech-in-noise task. Critically, these effects were observed under behavior-matched listening conditions, isolating longitudinal changes in cortical response magnitude from differences in task performance or perceptual accuracy. These changes were most prominent in the right-hemisphere auditory and perisylvian regions and were observed across task epochs and trial types. Taken together, these reductions across the auditory and perisylvian regions under behavior-matched listening conditions can be interpreted as experience-dependent modulation of cortical response magnitude following short-term hearing aid use.

To our knowledge, this is the first magnetoencephalography study to demonstrate such structured longitudinal modulation of source-level cortical responses following short-term hearing aid use in older adults with age-related hearing loss under behavior-matched conditions. Since task performance was explicitly matched across recording sessions, the observed reductions in normalized MEG magnitude are interpreted as reflecting changes in cortical response magnitude under equivalent listening demands rather than differences in perceptual accuracy. One plausible interpretation is that reduced cortical response magnitude following hearing aid use reflects reduced processing demands or altered neural recruitment during speech-in-noise listening. Importantly, these effects were not diffuse but showed a consistent and regionally structured pattern, most prominently involving right-hemisphere auditory and perisylvian regions. The emergence of such systematic changes after only 3 months of hearing aid use suggests that cortical responses during challenging listening conditions are modifiable over relatively short time scales, highlighting that these neural responses can change even in later adulthood.

Reductions in normalized MEG magnitude following hearing aid use were observed across both Noise and Sentence epochs, with the spatial distribution and statistical significance of these changes varying across regions of interest and trial types. Correct and incorrect trials were analyzed separately to describe the longitudinal patterns of response magnitude change, and no direct statistical contrasts were performed between trial outcomes. Accordingly, differences across trial types are described descriptively rather than interpreted as reflecting distinct functional or mechanistic processes. The inclusion of both trial types serves to characterize the robustness and context-dependence of longitudinal response changes across task segments rather than to support inferential claims about successful versus unsuccessful speech perception.

The Noise and Sentence epochs capture different segments of the task (Noise presented in isolation versus the concurrent presentation of Noise and speech) and therefore reflect distinct auditory contexts. The Noise and Sentence epochs capture different segments of the task (Noise presented in isolation versus the concurrent presentation of Noise and Speech) and therefore reflect distinct auditory contexts. Activity measured during the Noise epoch does not constitute a direct proxy for neural processing of background noise during speech-in-noise listening, which additionally involves processes related to concurrent signal integration and scene parsing. Nevertheless, longitudinal changes observed during the Noise epoch may reflect a more general modulation of cortical responsiveness to sustained acoustic stimulation following hearing aid use rather than a mechanism specific to noise suppression during speech perception. From this perspective, the observed reductions in normalized MEG magnitude across epochs and regions may reflect a redistribution or recalibration of cortical response magnitude (or gain) under equivalent listening demands, potentially related to altered sensory input statistics following amplification (Rabinowitz et al., 2011).

During correct trials, reductions in normalized MEG magnitude were observed in several right-hemisphere auditory and perisylvian regions, particularly during the Noise epoch. In contrast, during incorrect trials, reductions were more prominent during the Sentence epoch and primarily involved right-hemisphere regions. Since no inferential comparisons were conducted across trial outcomes or epochs, these observations are not interpreted as evidence of trial-specific neural mechanisms or differential processing efficiency. Instead, one plausible interpretation is that hearing aid use is associated with context-dependent modulation of cortical response magnitude, such that changes in sustained acoustic stimulation (Noise epoch) and concurrent speech-plus-noise processing (Sentence epoch) are reflected differently across task segments. Consequently, the observed differences across epochs and trial types may reflect variability in how cortical responses are redistributed under matched listening demands rather than mechanisms tied to successful or unsuccessful speech perception per se. Longitudinal changes in normalized MEG magnitude were therefore evident across multiple task segments and conditions, consistent with a general modulation of cortical response characteristics following short-term hearing-aid use.

This preliminary study does have some limitations that are worth addressing. The primary limitation is the small sample size, which limits the generalizability of certain findings and our ability to adequately control for multiple statistical comparisons. However, we believe that several trends emerge beyond individual test results when parsing the findings as a function of stimulus type, brain hemisphere, and SIN performance. A greater number of HINT trials should have been used to improve the signal-to-noise ratio, as this would have allowed us to analyze data from both conditions separately. The inclusion of an additional time point before the baseline measurements would enable the capture of a better baseline by determining the trajectory of activity change prior to the intervention. Similarly, the inclusion of a study time point after the intervention would have allowed us to determine the long-term stability of the observed changes. Finally, linguistic heterogeneity represents an additional limitation of the present study. Participants completed the speech-in-noise task in either English or French, depending on their native language. Although task structure and performance criteria were matched across languages, differences in phonological, prosodic, and lexical characteristics may influence speech-in-noise processing demands and associated cortical responses. The small sample size precluded language-stratified analyses, and future studies with larger, language-homogeneous cohorts will be needed to more directly assess the impact of linguistic factors on longitudinal neural responses following hearing aid use.

From a clinical perspective, the present findings suggest that cortical responses during speech-in-noise listening are sensitive to changes in auditory input over relatively short time scales following hearing aid adoption in older adults. Although the present study was not designed to assess clinical efficacy or behavioral outcomes, the observation of structured longitudinal modulation of cortical response magnitude under behavior-matched conditions indicates that the neurophysiological measures may provide a complementary window into how auditory interventions alter cortical processing demands over time. These findings may be particularly relevant when considered alongside evidence that user satisfaction and perceived benefit from hearing aids vary across individuals and may be influenced by subjective listening experiences and device-related factors (Portelli et al., 2025). Such measures may prove useful in future research aimed at characterizing individual variability in neural response to amplification, optimizing intervention strategies, or identifying neural markers of auditory system adaptation, without presupposing functional remediation or behavioral improvement.

In summary, this exploratory study shows that normalized MEG magnitude during a speech-in-noise task changed over a 3-month interval in older adults with age-related hearing loss following the initiation of hearing aid use under conditions in which task performance was explicitly matched across sessions. These changes were regionally structured and observed across task segments, suggesting that cortical responses during challenging listening conditions are modifiable over relatively short time scales in later adulthood. One plausible interpretation is that the observed longitudinal reductions in response magnitude reflect the experience-dependent modulation of auditory cortical engagement under altered sensory input conditions rather than the remediation of a pathological process. Accordingly, the present findings demonstrate that short-term hearing aid use is associated with reproducible, regionally structured changes in cortical response magnitude during speech-in-noise listening in older adults, highlighting the sensitivity of auditory cortical responses to altered sensory input even later in adulthood.

## Data Availability

The raw data supporting the conclusions of this article will be made available by the authors, without undue reservation.

## Footnotes

1
http://neuroimage.usc.edu/brainstorm
