# Supplementary material for: Short-term hearing aid use reduces auditory cortical responses to speech-in-noise listening among older adults with age-related hearing loss
Source: Front Aging Neurosci. 2026 Apr 16;18:1690956. doi: 10.3389/fnagi.2026.1690956 (PMC13130390; doi:10.3389/fnagi.2026.1690956)

**Supplementary Table 1**. Wilcoxon signed-rank test results for the noise and sentence epochs for correct trials.


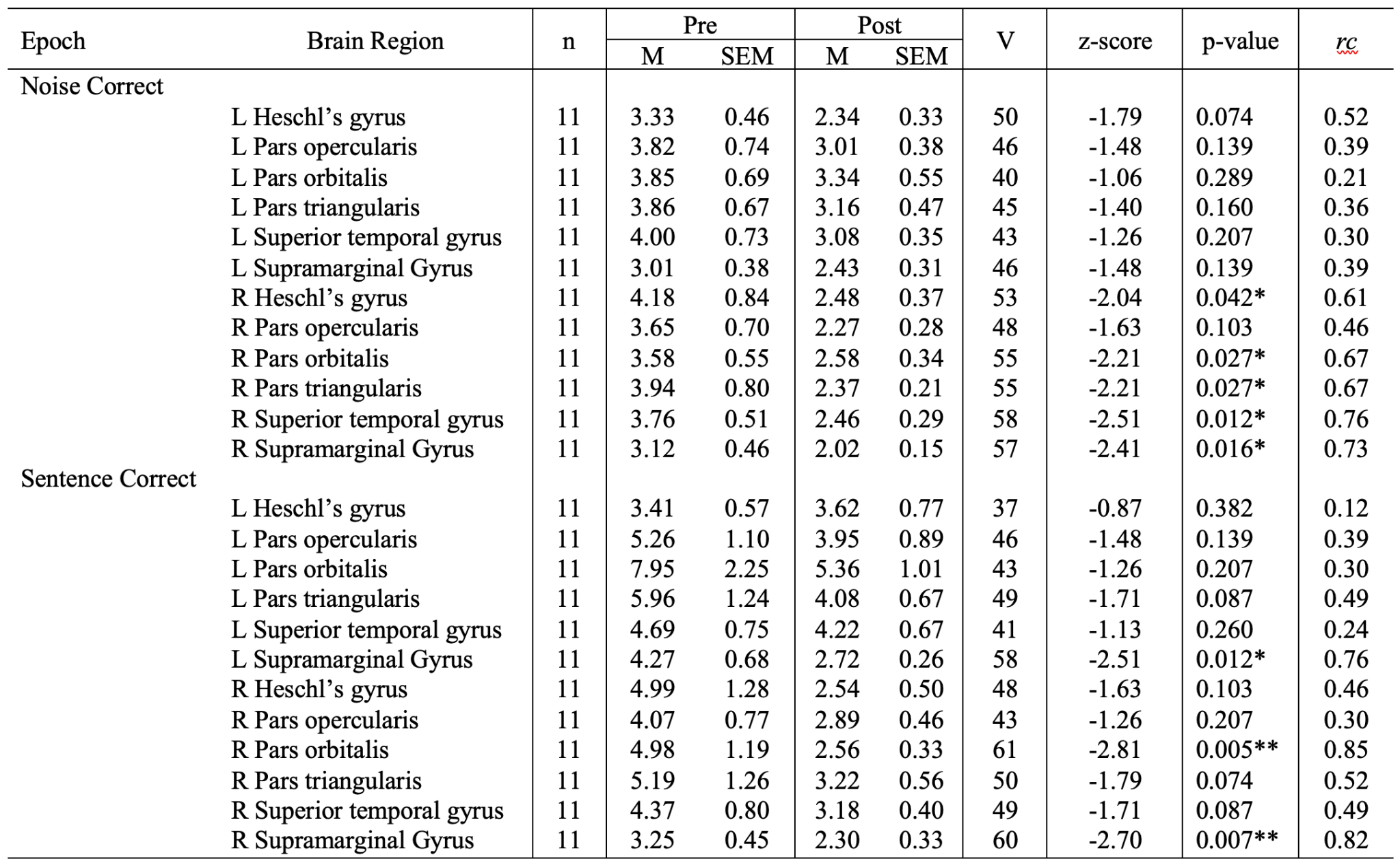


**Supplementary Table 2**. Wilcoxon signed-rank test results for the noise and sentence epochs for the incorrect trials.


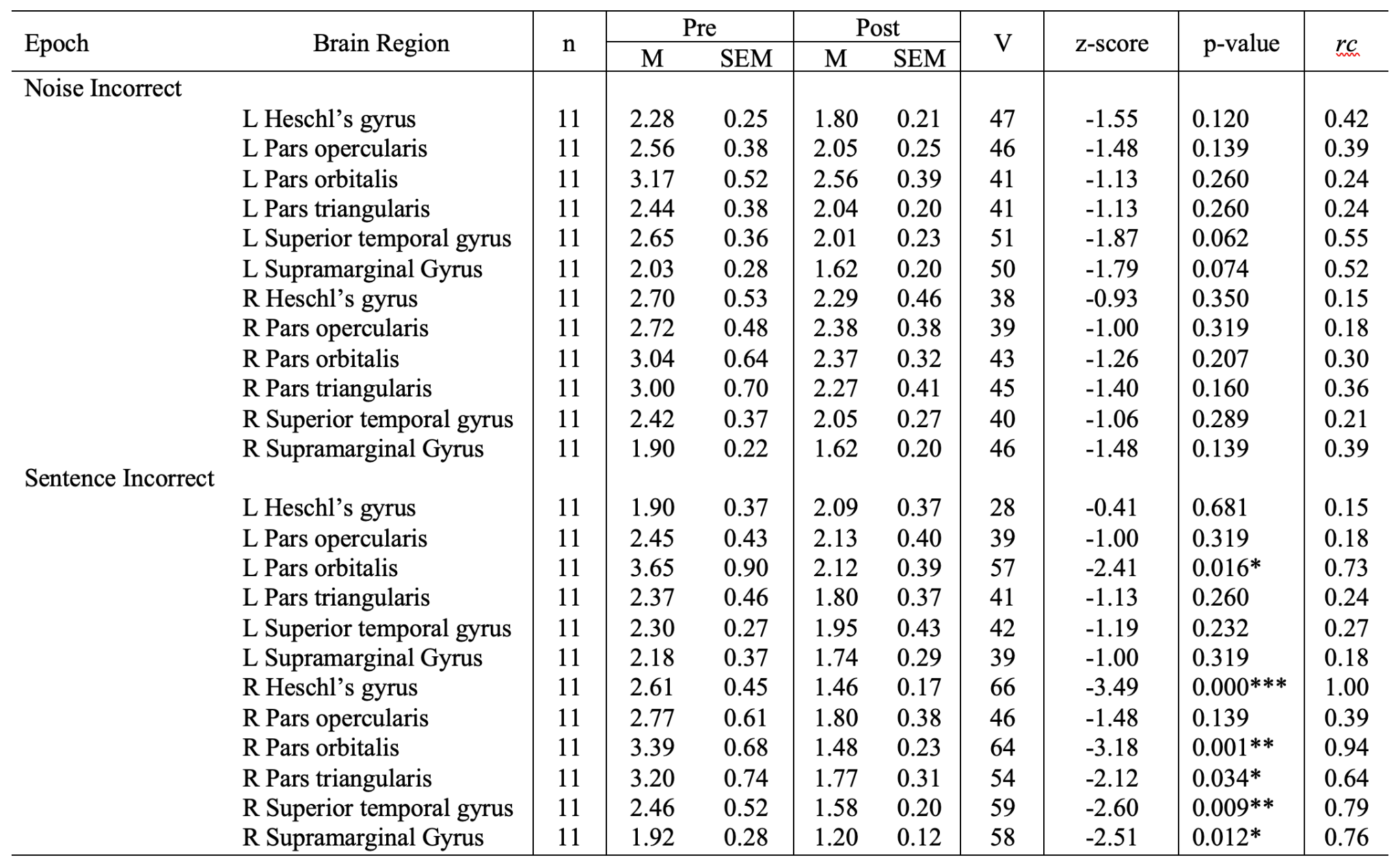

Supplement: Supplementary file 1 [file Data_Sheet_1.docx]
